# Gender Comparisons of Asymmetric Lateralization in Neurodegenerative Disorder Patients via E-Pen Based Cognitive Assessment System

**Published:** 2019-02

**Authors:** Haemi JEE, Jaehyun PARK

**Affiliations:** 1. Department of Sports and Health Care, Namseoul University, Cheonan-si, Korea; 2. Department of Information and Communication, Inha University, Incheon, Korea

**Keywords:** Line bisection test, Asymmetric neglect, Visuospatial neglect, Parkinson’s disease

## Abstract

**Background:**

The clinical gender-dependent characteristics of visuospatial neglect between men and women have not been elucidated in Korean patients with cognitive impairment. The goal of this study was to observe the asymmetric lateralization in patients using a novel e-pen based cognitive assessment system.

**Methods:**

A total of 31 patients, 16 men and 15 women, with early stage hemispheric cerebral dysfunction were recruited for the assessment of unilateral neglect suing a novice paper-and-pencil based electronic evaluation system from a rehabilitation center of Inha University hospital in 2016. Results were assessed for degrees of deviations, and numbers of neglected lines. Degree of deviation was assessed using the positions and distances from the horizontal line centers. Effect sizes were calculated to assess proximities between the assessed results.

**Results:**

Comparatively greater left and rightward biasness for the right-sided and left-sided horizontal lines were observed for the male patients, respectively. Moreover, greater degree of left to rightward biasness was observed as the horizontal lines shortened in both groups. However, the magnitude of biasness in female patients showed comparatively less directional bias, indicating greater prevalence for the center of mass effect in male patients.

**Conclusion:**

Gender difference in visuospatial neglect seems to exist with less accuracy in recognition for the bisecting center for the female and asymmetrical lateralization and magnitude of deviation for the male patients.

## Introduction

Cerebral impairment often exhibits motor dys-function of the contralateral side and potentially limitation for rehabilitation (1). Asymmetric motor dysfunction and cognitive impairment in patients with a degenerative brain lesion show greater display of inattention or reduced attention for biasness toward a particular side of the body. Unilateral or visuospatial neglect is presented in a spectrum of clinical disorders. Parkinson’s disease (PD) is one of the clinical disorders that may display unilateral neglect., and in PD, degeneration of dopaminergic cells in the substantia nigra pars compacta and consequential asymmetric dysfunction of the dorsal striatal projection area and posterior putamen have been described to explain motor asymmetry in PD patients (2, 3). Dopaminergic depletion in these sensorimotor areas may be involved in the abnormal motor and cognitive symptoms frequently delineated in PD and unilateral neglect (3, 4).

In general, asymmetric neglect is a common and disabling neuropsychological condition characterized by the inability to perceive contralesional stimuli due to damage of one of the cerebral hemispheres (5, 6). Patients with asymmetric neglect fail to recognize, identify, perceive, detect, respond to, or attend to stimuli from the side contralesional to the hemispheric lesion (1, 7–10). This inability to detect or respond to stimuli from the contralesional side leads to a greater rate of deviation and adversely affects daily activity and quality of life (7, 8, 11, 12).

Despite the similar unilateral manifestations and characteristics, the physiological mechanisms of unilateral neglect may differ in PD patients. Cognitive impairment and motor dysfunction may manifest as bradykinesia, rigidity, tremor, and/or attentional mismanagement. Patients may exhibit biased neglect toward ipsilateral, contralateral, or bilateral neglect despite predominant left-sided hemibody symptoms (13, 14). Despite the different speculations, it is unclear whether hemineglect in PD patients is related to impaired attentional control or impaired action-intention toward particular space due to motor dysfunction (14). Whereas impaired action-intention may influence asymmetric neglect, imbalanced focal and global spatial attentions have been suspected to be related to hemineglect in PD. Given a rapid increase in the elderly population in Korea, the prevalence of age-related diseases, such as PD, has also been increasing noticeably. Acknowledging the unilateral nature appears to be critical for developing improved rehabilitation programs for improving quality of life and outcome (15).

In particular, elucidating gender-related differences in the nature of visuospatial neglect may facilitate rehabilitation strategies in PD patients, as discrepant pathophysiological natures have been reported in male and female PD patients (10, 16). For example, a higher prevalence rate of PD (1.6-fold higher) has been reported in men (16). In addition to the increased prevalence of PD in men, different pathophysiologies have been reported (17, 32–34). In particular, gonadal hormone and chromosomal factors have been reported to be related to neuroprotective effects on the dopaminergic system, and longer exposure to estrogen has also been reported to reduce the risk of PD and provide protection against dopaminergic neuron loss during diseased and non-diseased states (16, 17). However, although some comparative studies reported greater disability and poorer quality of life in female patients, others have reported no gender difference (10).

Therefore, this study was conducted to observe and analyze unilateral neglect attributes in Korean male and female PD patients with partial cerebral deterioration and hemineglect (18). The existence and degree of perceptual neglect was assessed using an electronic pen (e-pen) based cognitive assessment system, which was developed specifically to access characteristics of unilateral neglect. The e-pen based system was designed to run the paper-and-pencil based line bisection test (LBT) to obtain assessment results of LBT time, number of deviated strokes by centered, left-sided, and right-sided locations, percent deviation, extend of deviation by location and length, and number of neglected lines. These results were obtained and analyzed and the results of male and female PD patients were compared.

## Methods

### Subjects

The experiment was conducted at a rehabilitation center of Inha University hospital in 2016. Thirty one PD patients were divided into two groups based on sex: 16 female patients and 15 male patients. The mean patient ages (SD) were 42.9 (±7.7) men and 41.2 (±7.5) years women, and education durations were 14.9 (±2.1) and 13.9 (±2.8) years, respectively. PD patients were diagnosed to have neurologic deficits at most 5 years before study commencement by physicians with more than 7 years of experience of neurology and rehabilitation. The study protocol and procedures were approved beforehand by the institutional medical ethics committee in accord with the ethical standards of the World Medical Association Declaration of Helsinki on Ethical Principles for Medical Research. After being given a detailed explanation of the study objectives and procedures each participant provided written informed consent.

The study inclusion criteria were as follows: 1) the ability to participate in a rehabilitation program and perform the LBT test, 2) the ability to follow verbal directions, 3) the ability to conduct self-care activities, 4) stable medicated, metabolic, and clinical states during the test, and the absences of 5) any neurological disease (other than PD) or active psychiatric disease, 6) a developmental or learning disability, and of 7) a major sensory deficit. Patients with an inconclusive diagnosis or health complication also were excluded.

### Experimental procedure

The assessment procedures were conducted by an examiner and a rater who were specialists with clinical background and experience in the field of neuropsychological rehabilitation for more than 2 years. Both the examiner and the rater were unaware of study details. The K-MMSE (Korean version of the Mini-Mental Status Examination) was conducted prior to the assessment of cognitive status. The K-MMSE scores of male and female patients were non-significantly different (29.1 (±1.2) and 29.0 (±1.7), respectively.

A novel electronic pen (e-pen) based cognitive assessment system composed of a line bisection test (LBT) paper, e-pen, and LBT program installed computer was prepared prior to the study. The novel e-pen based cognitive assessment system was developed to instantaneously transmit written or drawn information on a micro-pattern printed paper to a computer with installed LBT program. Bisecting horizontal lines on a LBT paper were drawn as previously described (7). Twenty horizontal lines were drawn parallel to the long axis on the paper of recommended size (21.5 × 28.0 cm) prior. Three 10, 12, 14, 16, 18, and 20 cm lines were drawn in-between two 15 cm centered horizontal lines drawn as references (7), and three horizontal lines of 6 different size horizontal lines were randomly assigned to center, left, and right horizontal lines. As the test began, each patient was provided with an e-pen and LBT printed micro-patterned paper by the examiner, who explained the test procedure. Test results were manually assessed accuracy by the rater. Prior to the e-pen based LBT test, patients were instructed on the test procedure by the clinical specialist. First, the patients were instructed to use the right hand and maintain the left hand on the table. Second, the patients were instructed to bisect each line by drawing a vertical line as close to the center of the horizontal line as possible. They were also instructed to draw only one bisecting line per a horizontal line and to bisect lines from top to bottom without skipping any line. Finally, patients were instructed not to move the LBT paper while conducting the test; the paper was fixed with tape to avoid excessive movement when needed. All participants used their dominant right hand while conducting the LBT (7). The LBT results were assessed in two ways. During the test, results were sent instantaneously to a computer for real-time calculation and results were displayed on a computer monitor. The LBT results were also blindly assessed by a rater specializing in the field of neurological rehabilitation and with more than two years of experience of clinical unilateral assessment. The rater counted the numbers and positions of neglected or unmarked lines and measured deviations from true centers using a metric ruler (cm), and assessed LBT results with a metric ruler, pen, and calculator (7). The two separate sets of results, that is, rater assessed and computer assessed results, were compared for reliability. Excellent reliability (*r*) of 0.92 (*P*<0.001) was obtained.

The distances (cm) from the centers of horizontal lines and deviations (%) were calculated. The total number of deviating strokes on each bisecting line was also counted. The deviated strokes were counted as either right or left deviations when placed ≥5% from horizontal line centers. Previous utilized formula (1/2 × length of horizontal line (cm) × 5%) was used to determine a meaningful deviation (7). Deviation (%) refers to the mean deviation score of the 18 bisecting scores obtained from bisecting the 18 horizontal lines for meaningful deviations (7). Distances (cm) from the centers were measured to the nearest 5 mm for comparisons. The center, left, and right bisected locations and the bisected horizontal lines with different lengths were compared. Neglected lines, that is, lines missed or completed out of order (top to bottom) were also counted.

### Electronic-pen based cognitive assessment system

**Fig. 1:**
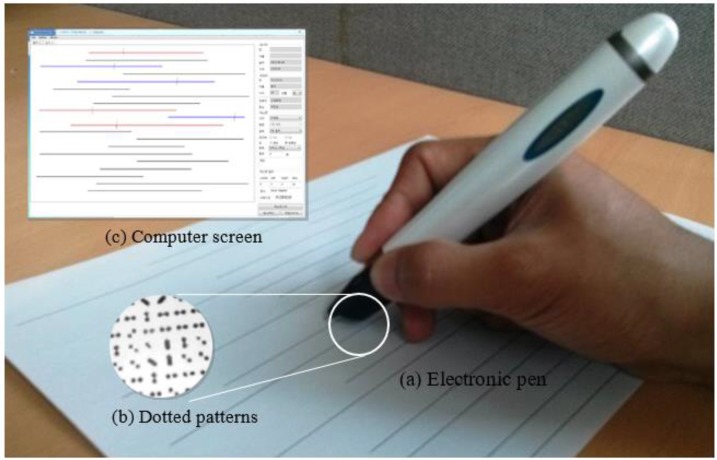
Electronic pen based cognitive assessment system

The based electronic-pen based paper-and-pencil cognitive assessment system was developed by the Embedded Computing Laboratory of Inha University (Incheon, Korea). This system was used to capture and record writing or drawing information in real-time (Fig. 1). The e-pen based system is a semi-computerized system that utilizes normal writing or drawing tools, such as, pen and paper, and computerized tools. The electronic pen system is composed of a position pattern recognizing (a) electronic pen (e-pen), (b) micro-pattern printed paper, and (c) an LBT computer software.

The e-pen system has been shown to high reliability and accuracy in clinical application (19). When the e-pen is pressed on the pattern printed paper, a pressure sensor senses the pressure and activates the reading device within the e-pen for real-time transmission of distinct located printed pattern information to a computer with the LBT program. Dotted images are acquired through the e-pen’s CMOS sensor with built-in optical filter and lens for high speed DSP processed. Specific e-pen locations were uniquely coded using various compositions of 16 to 25 dots sized between 50 and 80 um. One identifiable figure can be created with paired dots for initial location identification. In the present study, four different figures were grouped to make one mark for final location identification. The location codes generated can be used to express about 2 billion locations. The e-pen location information is acquired and transmitted 85 times per second via Bluetooth and provides accurate and realistic representations of written or drawn information. The e-pen system has the advantage of assessing results more accurately than the traditional human assessment method (19). The e-pen system was used in the present study to allow more accurate comparisons of results.

### Statistical Analysis

The study sample size was determined using data from previous studies (n ≥ 10) that used the line bisection test (8, 20, 21). Prior to analytical assessments of data sets, the normality analysis was conducted using the Kolmogorov-Smirnov test. Data were found to be normally distributed. One way ANOVA (analysis of variance) was conducted to determine the significances of differences between the male and female groups.

The effect sizes (R^2^) of the mean differences were also calculated to present the relationship between the groups (Cohen’s D). Effect sizes between 0.2 to 0.3 is considered to have a ‘small’ effect, around 0.5 a ‘medium’ effect and 0.8 to infinity, a ‘large’ effect (22, 23). The data were analyzed with MedCalcR statistical software, Version 12.0 (Mariakerke, Belgium). Results are presented as means ± standard deviations (SD). Statistical significance was accepted for the *p*-value of less than 0.05 and 95% confidence intervals.

## Results

The two groups were compared for the LBT duration, neglected lines, and percent deviations. Effect sizes of 0.22, −0.05, and 0.02 were first calculated for the LBT duration, neglected line number, and percent deviation, respectively (Table 1). The left and right-sided horizontal lines were counted. Numbers of strokes with a deviation of <5% from actual centers of horizontal lines were counted, and categorized as right or left deviations. Obtained effect sizes were −0.05, −0.01, and −0.04 for centered, left deviated, and right deviated strokes between the groups, respectively (Table 1).

**Table 1:** Results of the line bisecting test - LBT duration, percent deviation, and number of strokes and neglected lines

***Variable***	***Males Mean (SE)***	***Females Mean (SE)***	***Mean diff. (SE)***	***Confidence Interval***	***Effect size (R^2^)***
LBT duration (sec)	59.64 (±6.83)	54.89 (±4.04)	4.74 (±7.81)	−11.24 – 20.73	.22 (−.49 – .92)
Percent deviation (%)	.02 (±.05)	.01 (±.054)	.00 (±.07)	−.14 – .15	.02 (−.70 – .73)
Number of centered strokes	6.64 (±1.58)	6.94 (±1.57)	−.30 (±2.23)	−4.86 – 4.27	−.05 (−.77 – .67)
Number of deviated strokes toward left	7.27 (±1.58)	7.31 (±1.42)	−.05 (±2.12)	−4.37 – 4.29	−.01 (−.71 – .70)
Number of deviated strokes toward right	5.57 (±1.03)	5.75 (±1.30)	−.18 (±1.70)	−3.65 – 3.29	−.04 (−.76 – .68)
Number of neglected lines	6.64 (±1.57)	6.94 (±1.57)	−.29 (±2.23)	−4.86 – 4.27	−.05 (−.77 – .67)

SE: standard error of mean, R^2^: coefficient of determination

**Fig. 2A and 1B:**
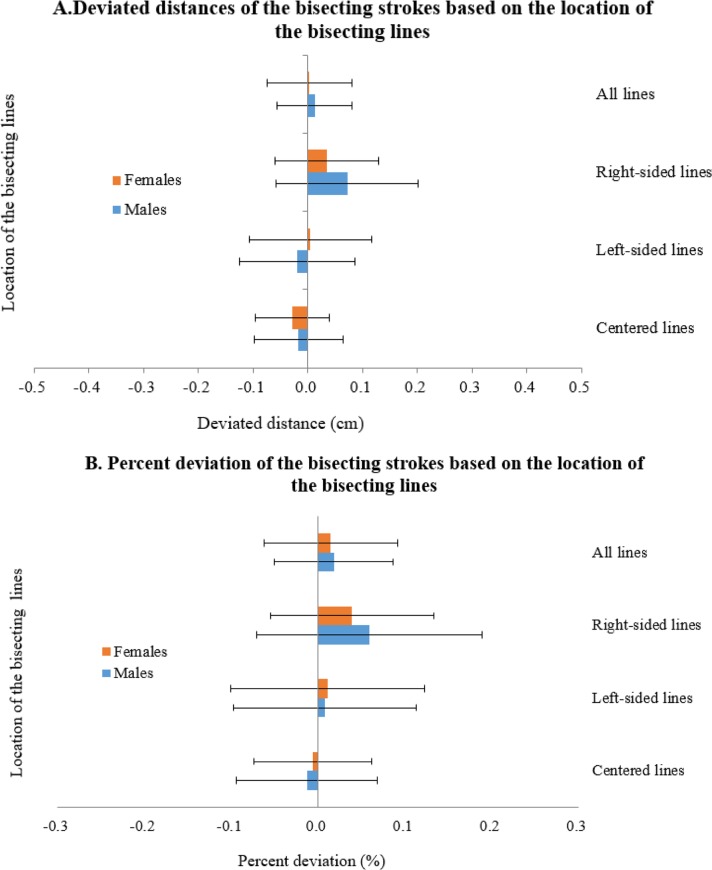
The degree of deviation of the bisecting strokes according to the location of bisecting lines on the line bisection test paper expressed in distance and percent deviation All lines: mean and standard error of mean (SE) for 16 bisecting lines; right-sided lines: mean and SE for 6 right-sided bisecting lines; left-sided lines: mean and SE for 6 right-sided bisecting lines; centered lines: mean and SE for 6 centered bisecting lines; percent deviation (%): 1/2 × length of bisecting line × 5 %

Degrees of deviation from the centers of horizontal lines were calculated using the overall, right-sided, left-sided, and centered locations and by lengths of 10, 12, 14, 16, 18, and 20 cm to compare the two groups. The number of bisected strokes and deviated distances from actual centers were calculated as distances (cm) and deviations (%) (Fig. 2A and 2B).

For women, the bisecting strokes deviated by −0.032, −0.004, and 0.033 cm from actual centers of centered, left-sided, and right-sided horizontal lines, respectively, with an overall average deviation of .003 cm. For men, the bisecting strokes deviated by −0.010, −0.013, and 0.072 cm from actual centers for centered, left-sided, and right-sided bisecting lines, respectively, with an overall average deviation of 0.012 cm. The magnitudes of deviated strokes were also assessed; for men, the degrees of deviations (%) were −0.011, 0.014, and 0.062% for centered, left-sided, and right-sided lines, respectively, with overall deviation of 0.021%, and for women these were −0.014, 0.012, and 0.041%, respectively, with an overall deviation of 0.01%.

**Fig. 3A and B:**
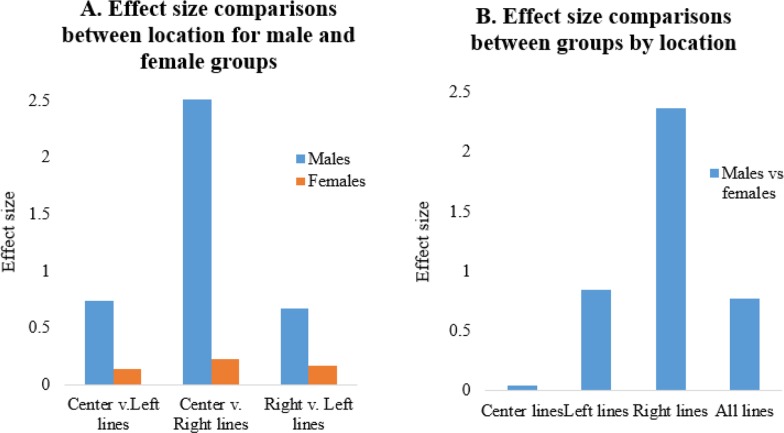
Effect size comparisons of the bisecting lines between the male and female patients by location and gender Center: centered bisecting lines; Left: left-sided bisecting lines; Right: right-sided bisecting lines; effect size: Cohen’s d

The effect sizes of deviated strokes were also compared for centered, left-sided, and right-sided horizontal lines for men and women (Fig. 3A and 3B). The effect sizes of deviated bisecting strokes by distance (cm) were 0.042, 0.846, and 2.371 for centered, left-sided, and right-sided horizontal lines, respectively, between the male and female patients with an overall effect size of 0.77 (Fig. 3A). The locations of bisecting lines were also compared. The effect sizes between the center and left, center and right, and right and left horizontal lines were .741, 2.542, and 0.678, respectively, for men, and 0.141, 0.227, and 0.163, respectively, for women (Fig. 3B).

**Fig. 4A and B:**
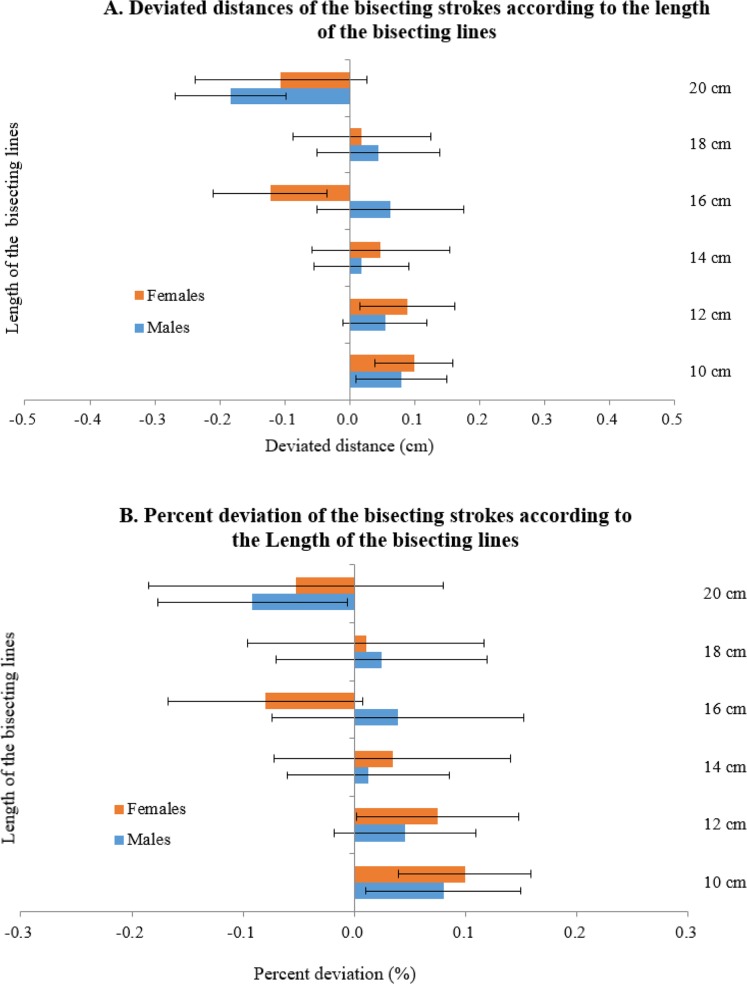
The degree of deviation of the bisecting strokes according to the length of the bisecting lines on the line bisection test paper expressed in distance and percent deviation

Lastly, the degrees of deviation of bisecting strokes were calculated based on the length of raw horizontal lines for men and women (Fig. 4A and 4B). Absolute distances (cm) for horizontal lines of length 10, 12, 14, 16, 18, and 20 cm were 0.083, 0.051, 0.024, 0.065, 0.052, and −0.186 cm, respectively, for men and 0.101, 0.091, 0.05, − 0.122, 0.024, and −0.117 cm for women, respectively (Fig. 4A), and the percent deviations (%) for the bisecting line lengths of 10, 12, 14, 16, 18, and 20 cm were 0.087, 0.051, 0.012, 0.044, 0.023, and 0.096 %, respectively, for men and 0.101, 0.076, 0.031, −0.088, 0.017, and −0.056 %, respectively, for women (Fig. 4B).

## Discussion

Asymmetric or visuospatial neglect is presented in patients with neurological dysfunctions or traumatic injury to one side of the brain (7). Usually, patients with damage to one side of the cerebral hemisphere perceive stimuli from contralesional sides. However, the tendency for unilateral neglect in patients with PD (Parkinson’s disease) has been reported to be comparatively unique and is associated with complications, such as, motor dysfunction and hemibody dominant symptoms (13). Moreover, symptoms may exhibit gender difference (24, 25). This study was conducted to compare and understand gender-associated differences in asymmetric neglect in Korean PD patients. Line bisection test (LBT) was conducted to observe the LBT duration, neglected line, and percent deviation. KMMSE (Korea Mini-mental State Examination) was also assessed to observe the cognitive state of the subjects.

Asymmetric dopaminergic depletion in PD affects cognitive pathways and diminishes neuro-psychological performances (4). Cognitive assessment is commonly performed to assess the degree of deterioration in patients with neurological disorder. In this study, the KMMSE results which were within the normal range showed no significance between the group. Although KMMSE has been used to test the cognitive state of PD patients in clinical settings, KMMSE has been reported to be less sensitive for the detection of early stages of cognitive dysfunction and to be inadequate for assessing areas of cognitive impairment, such as, executive function (26).

The line bisecting test (LBT) completion time may be related to the degree of impairment of executive function. In the present study, the LBT completion times were around one minute for male and female groups. The LBT durations of cognitively impaired stroke patients have been reported to be more than twice (106.8 (±28.81) seconds) that of cognitively healthy subjects (19). The KMMSE and LBT completion time results indicated that the cognitive deterioration state of the subjects were normal or at an early cognitive deterioration state.

The numbers of center, left, and right side bisecting lines were similar for the male and female patients. Although group difference was hardly shown, more strokes were drawn toward the left side of the horizontal lines by both groups. Moreover, the deviations were toward the left in both groups. Cognitively healthy adults also tend to draw bisecting lines slightly leftward, which is known as pseudoneglect (27). Previous report indicated that the demand for global attention may increase the right hemisphere activation and attention toward the left visuospatial area (14).

Although the number of bisecting strokes and marginally leftward deviations were similar to those observed in the cognitively healthy subjects, the degrees of deviations were divergent for the right-sided and left-sided horizontal lines. That is, the magnitude of the rightward deviations were greater (positive bisection error) than the leftward deviations for the male patients (Figures 1A and 1B), which is reminiscent of previous reports on the center of mass effect (14, 28). A greater tendency toward the rightward bisection may be observed for patients with PD since both the right and left hemispheres sub-serve global and focal attention, respectively (14). Furthermore, the left cerebral hemisphere impairment has been reported to reduce the ability to disengage focal attention, and right cerebral hemisphere impairment has been known to deactivate the ability to provide global attention (29). Global attention capability may be suppressed by a diminished ability to disassociate focal attention. Such suppressed capability may have induced greater right-ward deviation on the right-sided horizontal lines. Such a hypothesis appears possible since the reduced ability to disengage focal attention is mediated by the left cerebral hemisphere (29). The large effect sizes of right-sided lines observed in the present study strengthen this hypothesis (Fig. 2A).

Greater rightward bias for the right-sided horizontal lines suggests the center of mass effect. Greater number of patients with left hemibody symptoms may have influenced greater rightward bisection asymmetry. Therefore, different lengths of the horizontal lines were compared for deviating direction and magnitude (Fig. 3A and B). The effects of the lengths of horizontal lines were compared. Although deviation directions were mixed for longer lines, the deviations tended toward the right sides as the horizontal lines shortened. The center of mass effect seemed to increase as the horizontal lines were comparatively shorter and more visible. In healthy subjects, marginally leftward deviation is normal, especially for short horizontal lines. It has been suggested that the innate relative strength of the right hemisphere or left-to-right reading habit is responsible for dominant leftward bias (30).

The shift from one side to another in horizontal lines may be explained by the cross-over effect. Previous study which analyzed causes of unilateral proposed that a reflexive compensatory eye displacement fixates toward the contralesional space for short lines (30). On the other hand, attentional strength is strong enough to shift attention to the contralesional space as a counterbalance mechanism (30). In contrast to the attentional difference in spatial neglect, distortional representation of space was also proposed. Regardless of the mechanism responsible for the shift from right to left with increase in the horizontal line length, a progressive and consistent pattern was observed for both groups.

In terms of comparisons between differently sized horizontal lines (10 to 20 cm), women showed larger variations than men (Fig. 3A and B). However, women showed smaller effect sizes (< .03) in directional horizontal line comparisons (centered, left-sided, right-sided) (Fig. 2A and B). Such results indicate larger variations in degrees of deviations and less directional bias in women. Although women bisected lines farther away from actual centers, they showed less distortion and asymmetrical lateralization as determined by LBT (19).

## Conclusion

Gender difference in visuospatial neglect seems to exist between the male and female PD patients. Less accuracy in recognition of the centers of the bisecting lines was shown in the female patients. On the other hand, asymmetrical lateralization and magnitude of deviation may be more prevalent for the male PD patients.

## Ethical considerations

Ethical issues (Including plagiarism, informed consent, misconduct, data fabrication and/or falsification, double publication and/or submission, redundancy, etc.) have been completely observed by the authors.
